# MR–CT image fusion method of intracranial tumors based on Res2Net

**DOI:** 10.1186/s12880-024-01329-x

**Published:** 2024-07-08

**Authors:** Wei Chen, Qixuan Li, Heng Zhang, Kangkang Sun, Wei Sun, Zhuqing Jiao, Xinye Ni

**Affiliations:** 1 School of Computer Science and Artificial Intelligence, Changzhou University, Changzhou, 213164 China; 2 Department of Radiotherapy, The Affiliated Changzhou NO. 2 People’s Hospital of Nanjing Medical University, Changzhou, 213003 China; 3 Jiangsu Province Engineering Research Center of Medical Physics, Changzhou, 213003 China; 4 Center for Medical Physics, Nanjing Medical University, Changzhou, 213003 China; 5 School of Microelectronics and Control Engineering, Changzhou University, Changzhou, 213164 China

**Keywords:** Intracranial tumor, Image fusion, Target delineation, Multiscale feature

## Abstract

**Background:**

Information complementarity can be achieved by fusing MR and CT images, and fusion images have abundant soft tissue and bone information, facilitating accurate auxiliary diagnosis and tumor target delineation.

**Purpose:**

The purpose of this study was to construct high-quality fusion images based on the MR and CT images of intracranial tumors by using the Residual-Residual Network (Res2Net) method.

**Methods:**

This paper proposes an MR and CT image fusion method based on Res2Net. The method comprises three components: feature extractor, fusion layer, and reconstructor. The feature extractor utilizes the Res2Net framework to extract multiscale features from source images. The fusion layer incorporates a fusion strategy based on spatial mean attention, adaptively adjusting fusion weights for feature maps at each position to preserve fine details from the source images. Finally, fused features are input into the feature reconstructor to reconstruct a fused image.

**Results:**

Qualitative results indicate that the proposed fusion method exhibits clear boundary contours and accurate localization of tumor regions. Quantitative results show that the method achieves average gradient, spatial frequency, entropy, and visual information fidelity for fusion metrics of 4.6771, 13.2055, 1.8663, and 0.5176, respectively. Comprehensive experimental results demonstrate that the proposed method preserves more texture details and structural information in fused images than advanced fusion algorithms, reducing spectral artifacts and information loss and performing better in terms of visual quality and objective metrics.

**Conclusion:**

The proposed method effectively combines MR and CT image information, allowing the precise localization of tumor region boundaries, assisting clinicians in clinical diagnosis.

## Introduction

Intracranial tumors are malignant growths that occur within the cranial cavity. They can be categorized into primary and secondary intracranial tumors according to their sites of origin [1]. Patients with intracranial tumors often experience severe central nervous system dysfunction, and typical clinical symptoms include headaches, vomiting, and dizziness. In some severe cases, patients may even suffer from stroke [2]. The primary treatment modalities for intracranial tumors include surgery, radiation therapy, chemotherapy, targeted therapy, and immunotherapy. According to the NCCN guidelines [3], radiation therapy is one of the primary treatment options for patients with various types and stages of intracranial tumors. It not only targets tumors directly but also alleviates intracranial symptoms, improves the quality of life, and extends the survival time for patients.

With the continuous advancement of medical imaging technology, multimodal image fusion techniques have become increasingly important in tumor target delineation [4]. Combining information from different modalities of images allows for the precise localization of tumor boundary regions, aiding doctors in accurately delineating tumor target areas. The accuracy of target delineation often depends on image quality and the experience of the medical professionals. High-quality images can enhance the ability of less experienced doctors to identify tumors, thus improving the precision of target delineation.

In the CT and MR images of patients with intracranial tumors, CT images primarily reflect electron density information of the skull and various intracranial regions. For structures with high electron densities, such as the skull, corresponding pixel values are high, resulting in bright regions in images. However, areas with low electron densities, such as tumors and soft tissues, have low pixel values, leading to dark signals. Thus, differentiating between lesions and surrounding normal tissues, especially small or early-stage lesions [5]. In contrast to CT, MR offers high resolution and rich soft tissue information. MR pixel values reflect the relaxation times of different tissue regions under a magnetic field, enabling it to capture clearer lesion boundaries and details than CT [6]. In clinical practice, the delineation of target areas in patients with intracranial tumors typically require the combination of MR and CT images [7]. Given that CT images have lower resolutions for soft tissue imaging, delineating intracranial tumor target areas based solely on CT images is challenging and requiring doctors to have extensive clinical experience. Compared with CT images, MR images can provide better soft tissue contrast, aiding in determining the relationship between tumor edges and surrounding normal tissues. Therefore, MR images are often combined with CT images to assist in delineating tumor target areas. In existing research and applications, MR–CT fusion images effectively improve the precision of intracranial tumor target delineation, enhance the accuracy of radiation therapy, and reduce radiation damage to surrounding normal tissues [8].

Currently, methods for MR–CT image fusion mainly rely on deep learning approaches [9]. These methods aim to address the challenge of effectively fusing features with different distributions and scales while preventing information loss and conflict. However, these algorithms often suffer from issues, such as poor texture detail in fused results and blurred boundaries [10]. These problems primarily stem from inappropriate feature extraction methods and fusion strategies. In many instances, feature extraction can lead to information loss because it focuses on single-scale features, such as local details and texture information, which have limited information representation capabilities. In medical image processing, multiscale features are widely used due to their strong information representation capabilities. They allow feature extraction at different scales to capture information within different spatial ranges [11].

Residual–Residual Network (Res2Net) is a feature extraction network known for its strong multiscale feature representation capabilities in recent years. It enhances model expressiveness by introducing multiscale attention mechanisms and utilizes residual connections to handle multiscale information, thereby avoiding information loss and inconsistencies between different branches. To improve the quality of fused images, this paper employs Res2Net in feature extraction to extract more scales and fine-grained features. Additionally, it proposes a spatial mean attention fusion strategy that generates different fusion weights for CT and MR images. The goal is to provide the fused image with detailed information and clearer boundary contours. Therefore, the main contributions of the proposed fusion method in this paper are as follows:

- Embedding Res2Net into the feature extractor is aimed at extracting finer-grained multiscale detail features more effectively. Additionally, employing Res2Net for image fusion results in fast fusion speeds.
- A fusion strategy based on spatial mean attention was designed, adaptively adjusting the fusion weights of feature maps to enhance the quality of the fused images.
- A hybrid loss function combining structural similarity loss and pixel loss was utilized to train both the feature extractor and feature reconstructor, aiming to preserve the texture and structure of the source images.

## Related work

### Medical image fusion

For medical image fusion, the classical fusion algorithm is based on multiscale transform (MST) and sparse representation (SR). MST decomposes an original image into multiscale layers and uses different rules to fuse decomposed multiscale layers. Finally, a fusion image can be obtained through multiscale inverse transformation. MST includes wavelet fusion [12, 13], pyramid [14, 15], Non-Subsampled Shearlet transform, and Non-Subsampled Contourlet transform [16, 17]. These algorithms can fully use multiscale information and select appropriate fusion rules for image features. However, multiscale operation requires decomposition, in which the number of decomposition layers is difficult to determine. The goal of SR is to generate fusion images from an overcomplete dictionary learned from a set of training images combined with a series of sparse coefficients. Li et al. [18] use SR and neighborhood energy activity operators to divide source images into base and detail layers and carry out feature fusion at different levels. This method is suitable for the fusion of gray level and color images. Liu et al. [19] proposed an image fusion method based on MST and SR, which combined the multiscale characteristics and adaptability of SR. However, the level of complexity of its time and space are higher than that of the time and space of a single MST or SR algorithm. A traditional method generates the weight graph by designing fusion rules manually, combines weight mapping with a fusion strategy, and finally generates a fusion result through inverse transformation. However, the fusion effect is not ideal because the fusion rules and decomposition methods of the design are complicated and laborious and the application scenarios are diverse.

With the continuous advancement of deep learning, many deep learning-based fusion methods have been widely proposed in recent years to effectively address the shortcomings of manual feature extraction, which often leads to insufficient representational capacity. In 2017, an unsupervised deep learning fusion algorithm called DeepFuse [20] was introduced, which significantly enhanced the efficiency and quality of fusion. However, it was primarily designed for multi-exposure image fusion. Li et al. [21] proposed a multimodal medical image fusion method based on CNN and supervised learning, enabling the fusion of different modalities in batch processing mode. Lahoud et al. [22] proposed a real-time medical image fusion method that utilizes a pretrained model to generate fused images containing features from multiple modal sources. However, although the fused images have clear textures, they contain noise that did not exist in the original images. Zhang et al. [23] introduced a CNN-based end-to-end fusion framework that can be directly applied to fuse CT and MRI images. Xu et al. [24] presented a unified unsupervised fusion network that adaptively updates preserved information through feature extraction and information metrics. Moreover, elastic weight consolidation algorithms for multiple fusion tasks were applied during network training, adjusting parameters based on new tasks while not forgetting previous tasks. Zhang et al. [25] proposed an end-to-end multitask fusion framework based on gradient and intensity ratio preservation, unifying the image fusion problem as a ratio problem between source image gradients and intensities. However, these models are specifically designed to provide a universal image fusion framework applicable to various tasks, thus overlooking the uniqueness of medical image fusion tasks, failing to fully represent the semantic information and visual features of multimodal medical images, and resulting in low-quality medical image fusion. Ma et al. [26] proposed a dual-discriminator conditional generative adversarial network (DDcGAN), which is suitable for medical image fusion at different resolutions. However, this model is aimed at fusing medical images of different resolutions and performs poorly on medical images of the same resolution. Owing to the powerful and versatile fitting capabilities of deep learning, it has enormous potential in the medical field, including applications, such as disease detection [27], lesion segmentation [28], disease classification [29], and surgical planning. Therefore, deep learning–based image fusion algorithms are expected to continue to emerge and be applied to the field of medicine.

### Res2Net

**Fig. 1:**
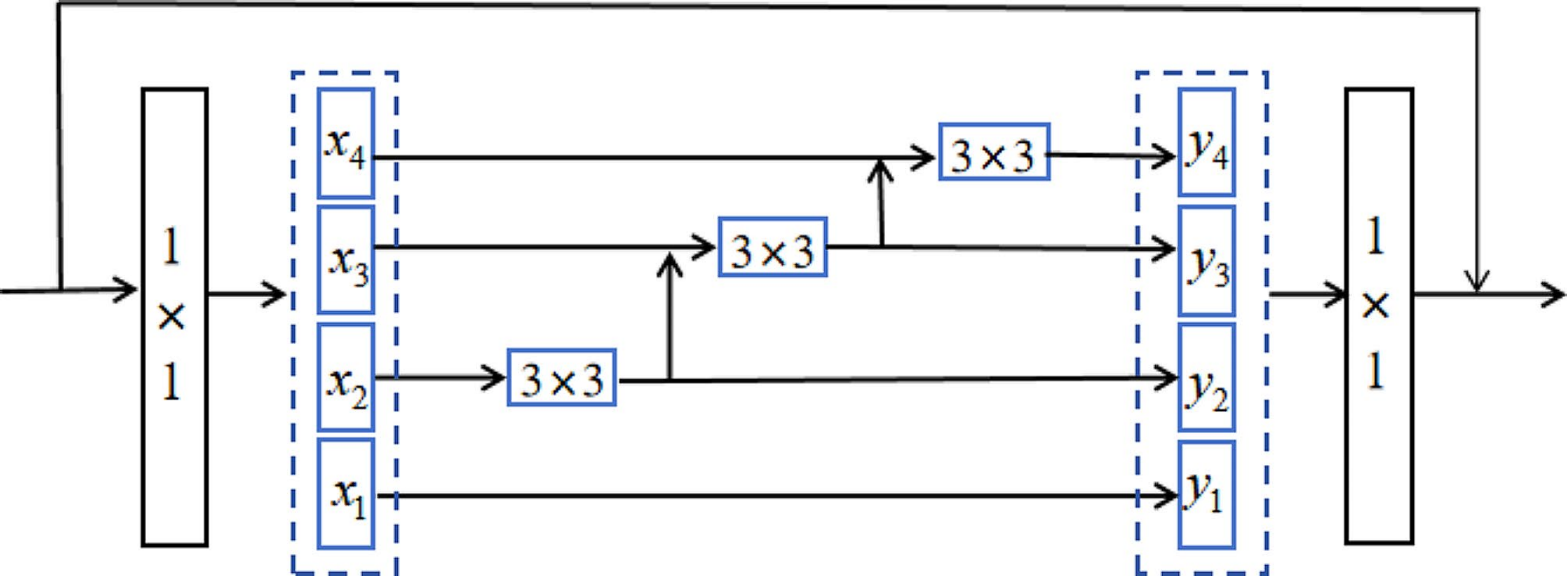
Res2Net structure

In the field of image processing, the purpose of feature extraction in deep learning models is to map sample sets from high-dimensional feature spaces to low-dimensional feature spaces and make the mapped sample sets to have good separability. The detail effect of feature extraction directly affects the quality of a whole model algorithm. The extensive comparative experiments of Geiros et al. [30] demonstrated that CNNs effectively extract texture details from original images. Therefore, we introduce an appropriate CNN module to extract texture details from original images, aiming to obtain better feature representations. In computer vision tasks, multiscale feature representations play a crucial role. Currently, most CNN-based medical image fusion algorithms do not consider multiscale feature representations or only conduct shallow multiscale feature representations, leading to considerable feature loss during feature extraction. Xu et al. [31] proposed an end-to-end fusion framework that incorporates unique information from different modal images by enforcing surface and deep constraints during model training. However, a single-plane fusion network model tends to ignore multiscale information from original images, resulting in an inadequate representation of fused image details. Li et al. [32] introduced a multiscale enhancement fusion network (MSENet) based on unique feature guidance, utilizing a dense three-path dilated network to enlarge the receptive field for the extraction of multiscale features. Song et al. [33] proposed a multiscale DenseNet (MSDNet), employing three filters of different sizes to extract multiscale features. However, MSENet and MSDNet acquire multiscale features by stacking network layers, resulting in the incomplete and inaccurate representations of multiscale features; thus, they are unable to fully achieve true multiscale feature representation. Therefore, at Pattern Analysis and Machine Intelligence 2020, Gao et al. [34] introduced a novel CNN module called Res2Net to address the limitation to multiscale feature representation capability. Res2Net is a network structure that combines multiscale attention mechanisms, aiming to handle the modeling of multiscale information and extraction of multilevel features. The Res2Net module is shown in Fig. 1.

In the Res2Net module, input features first pass through a 1 × 1 convolution for the production of a feature map, which is divided into n groups and denoted as ${x}_{i },i?\left\{\text{1,2},\cdots ,n\right\}$. Except ${x}_{i }$, each set of feature maps undergoes 3 × 3 convolution, the convolution operation is called ${H}_{i}(\bullet )$. An ${H}_{i}(\bullet )$output is denoted by ${y}_{i}$, the specific calculation process is shown in Formula (1)

1 $${y}_{i}=\left\{\begin{array}{c}{x}_{i} i=1\\ {H}_{i}\left({x}_{i}\right) i=2\\ {H}_{i}({x}_{i}+{y}_{i-1}) 2<i\le n \end{array}\right.$$

The n groups of ${y}_{i}$ are concatenated along the channel dimension before 1 × 1 convolution operation. In the convolution operation ${H}_{i}(\bullet )$ of group i an input contains multiple sets of input features. Therefore, Res2Net can extract fine-grained, multiscale features, effectively capturing global and local features.

## Methods

In this section, a detailed explanation is provided for the network model, fusion strategy, and loss function.

### Network model

DenseFuse [35] adopts the concept of dense connections to manipulate features at various scales, preserving abundant semantic information and texture details. By contrast, Res2Net enhances its feature extraction by incorporating attention mechanisms across different scales. This feature extraction approach captures feature information at various hierarchical levels, thereby enhancing the network’s capability to represent multiscale features. Consequently, the Res2Net model is employed for extracting multiscale features.

The input images of the model are CT and MR images, represented as ${I}_{ct}$ and ${I}_{mr}$, respectively. All input images are fused with pre-registration. The whole network structure includes feature extractor, fusion layer, and feature reconstructor. The feature extractor extracts multi-scale features from an input image and passes it into a fusion layer to obtain a multiscale feature fusion map. Finally, the fusion map is inputted into the reconstructor to reconstruct the image, and the fusion image ${I}_{f}$ is obtained. The architecture of the algorithm is defined as follows:

**Fig. 2:**
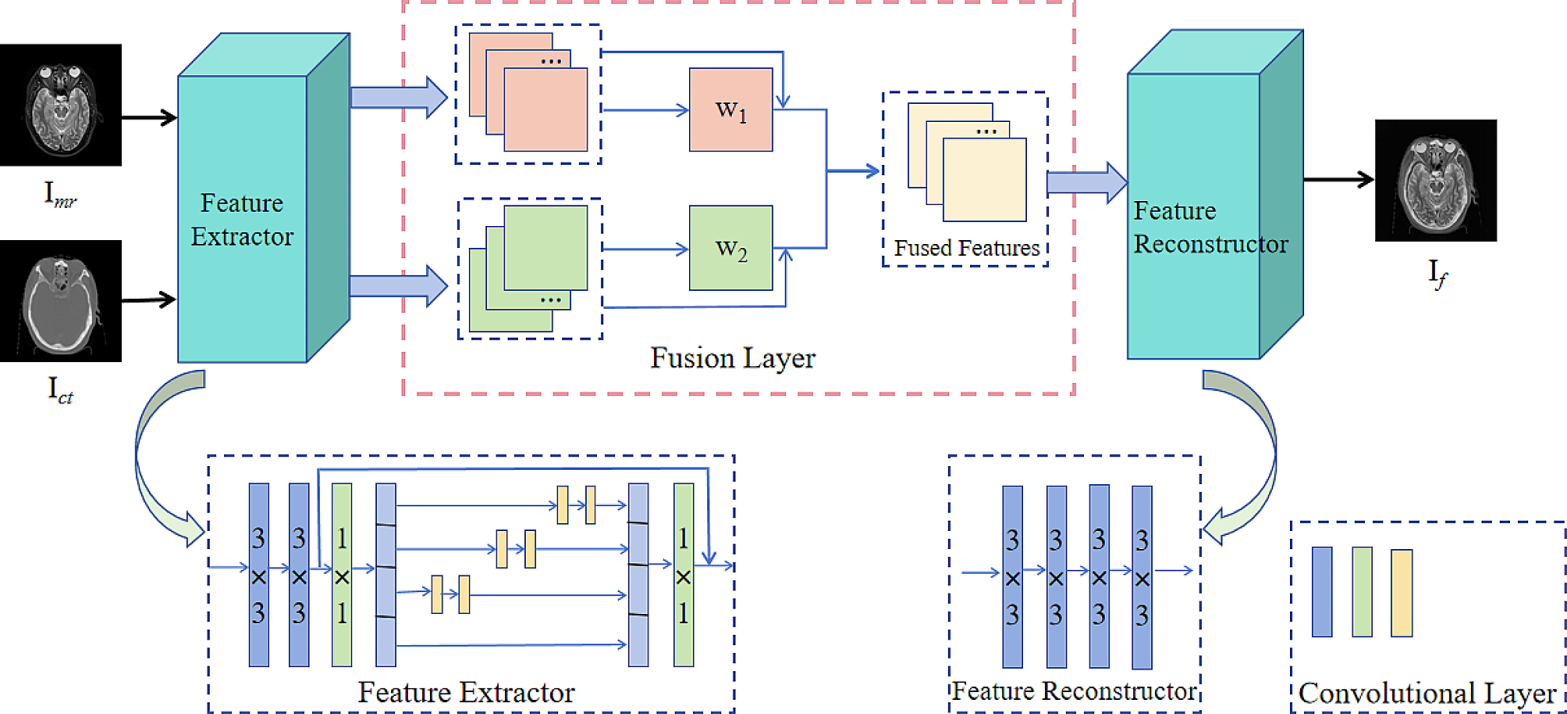
Architecture of proposed method

As shown in Fig. 2, the feature extractor consists of two 3 × 3 convolution filters and a Res2Net block. After the input features undergo two 3 × 3 and one 1 × 1 convolution operations, an input is fed to the Res2Net module to extract multiscale features. The operation and specific formulas for the Res2Net block are described in section B of the related work. The multiscale features extracted by Res2Net are transmitted to a fusion layer through 1 × 1 convolution. The feature reconstructor consists of four 3 × 3 convolution filters used to reconstruct the image. As shown in Table 1.

**Table 1:** Network structure of proposed method

	Layer	Size	Stride	Input Channel	Output Channel	Activation
Feature Extraction	Conv3	3	1	1	32	ReLU
	Conv3	3	1	32	64	ReLU
	Res2Net Block	-	-	-	-	-
Feature Reconstructor	Conv3	3	1	64	64	ReLU
	Conv3	3	1	64	32	ReLU
	Conv3	3	1	32	16	ReLU
	Conv3	3	1	16	1	-
Res2Net Block	Conv1	1	1	64	64	ReLU
	-	-	-	-	-	-
	Conv3 × 2	3	1	16	16	ReLU
	Conv3 × 2	3	1	16	16	ReLU
	Conv3 × 2	3	1	16	16	ReLU
	Conv1	1	1	64	64	ReLU

### Fusion layer

**Fig. 3:**
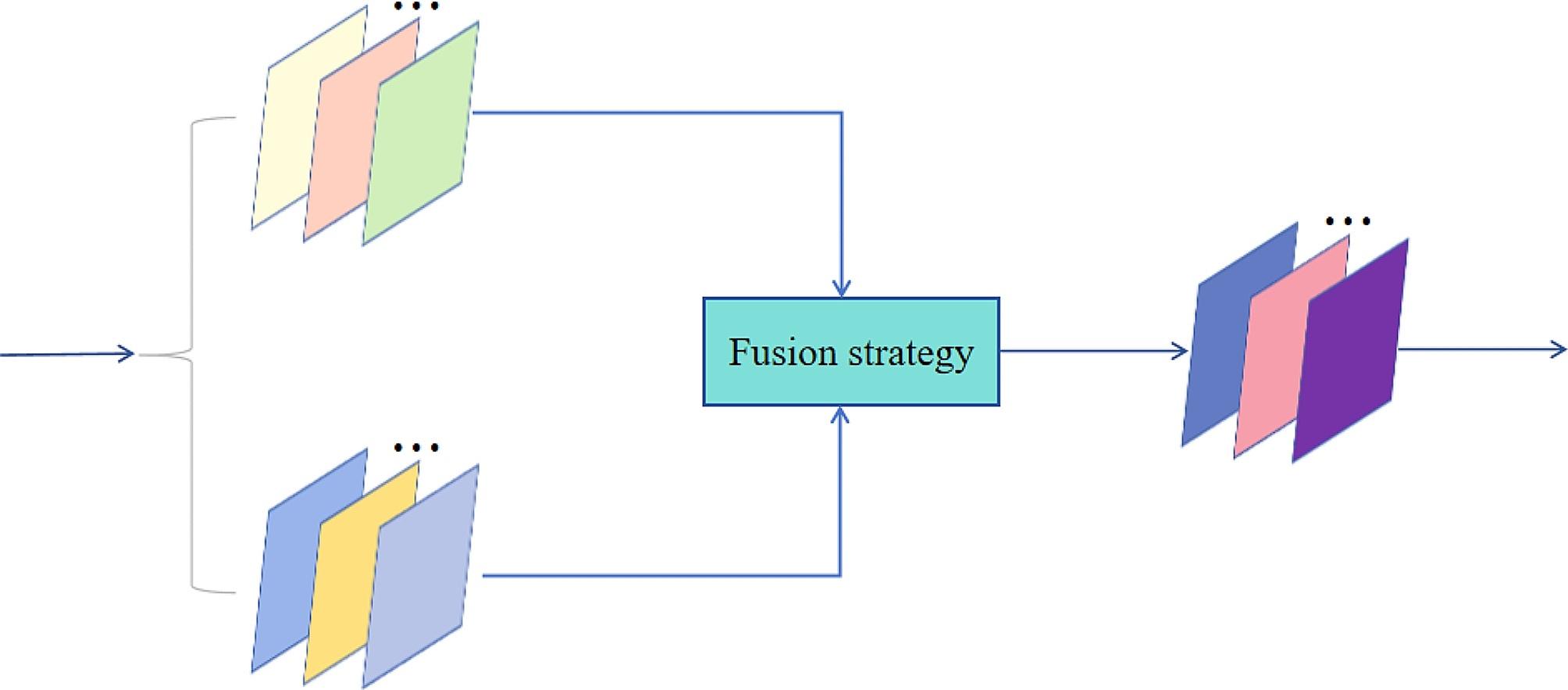
Fusion strategies

The main function of a fusion layer is to fuse extracted features (Fig. 3). Fusion strategy plays a pivotal role in image fusion, and the quality of fusion is closely related to a selected fusion strategy. The different modalities of images possess unique features, and suitable fusion strategies should be selected for different features. For MR and CT medical image features, a fusion strategy based on spatial attention mechanism is proposed, which can adaptively adjusts the fusion weights of feature maps according to difference between the average value and local average value at each position. Consequently, it preserves details and global structural features from source images.

1. Spatial Mean Attention Strategy.

Fusion weight is not considered in the fusion of addition strategy and average strategy. Thus, we propose a spatial attention fusion strategy based on mean operation. First, feature maps ${\varnothing }_{i}^{m}(x,y)$ extracted by the feature extractor is performed through means operation. Then, the soft-max operation is performed to calculate the weight maps ${\omega }_{1}$ and ${\omega }_{2}$. The formula is defined as follows:

2 $${\varvec{f}}^{m}=\sum _{i=1}^{s }{{\omega }_{i}\varnothing }_{i}^{m}\left(x,y\right)$$

3 $${\omega }_{i}(x,y)=\frac{Q\left({\varnothing }_{i}^{m}\right(x,y\left)\right)}{\sum _{i=1}^{s}Q\left({\varnothing }_{i}^{m}\right(x,y\left)\right)}$$

where, $Q\left({\varnothing }_{i}^{m}\right(x,y\left)\right)$ represents the mean operation of the position $(x,y)$ of each feature map and ${\varvec{f}}^{m}$ represents the fusion feature mapping obtained by the fusion layer. Finally, the fusion features are decoded and reconstructed by the ${\varvec{f}}^{m}$ input feature reconstructor, and the final fusion image is obtained.

### C. loss function

The structural similarity loss function helps maintain the structure and texture of an image during the generation process, resulting in realistic images. A pixel loss function aids in detail recovery and reconstruction, making the generated images closely resemble the pixel-level representation of real images. A hybrid loss function, denoted as *L*, which combines the structural similarity loss function and pixel loss function, is used to train the feature extractor and feature reconstructor, achieving more accurate reconstruction of input images. The specific definitions of the loss functions are as follows:

4 $$\varvec{L}={\varvec{L}}_{ssim}+{\varvec{L}}_{pixel}$$

${\varvec{L}}_{ssim}$ and ${\varvec{L}}_{pixel}$ are defined as follows:

5 $${\varvec{L}}_{ssim}=1-SSIM({\varvec{I}}_{fused}-{\varvec{I}}_{input})$$

6 $${\varvec{L}}_{pixel}=\frac{1}{BCHW}{\left|\right|{\varvec{I}}_{fused}- {\varvec{I}}_{input}\left|\right|}_{2}^{2}$$

where ${\varvec{I}}_{fused}$ and ${\varvec{I}}_{input}$ represent fused and input images, respectively, $B$ represents the batch size, $C$ represents the number of channels, and H and W represent the height and width of ${\varvec{I}}_{fused}$, respectively.

## Experiments and results

### Datasets and training details

The data set of this experiment consisted of brain image data of patients with nasopharyngeal carcinoma (1 MRI T1W sequence and CT information for each patient) collected in Changzhou Second People’s Hospital Affiliated to Nanjing Medical University from June 2018 to March 2021, aged 35–89 years old. MR Image obtained with a Philips Achieva Scanner 1.5T MR Device, T1W scanning parameters: TR1 343 ms, TE 80 msFA 90, image size 640 × 640 × 30–41, voxel spacing 0.6640 mm × 0.6640 mm × 5 mm. CT images were collected by GE Optima CT520 equipment. Scanning parameters were as follows: tube voltage 120 kV, tube current 220 mA, image size 512 × 512 × 101–123, voxel spacing 0.976 5 mm × 0.976 5 mm × 3 mm.

**Fig. 4:**
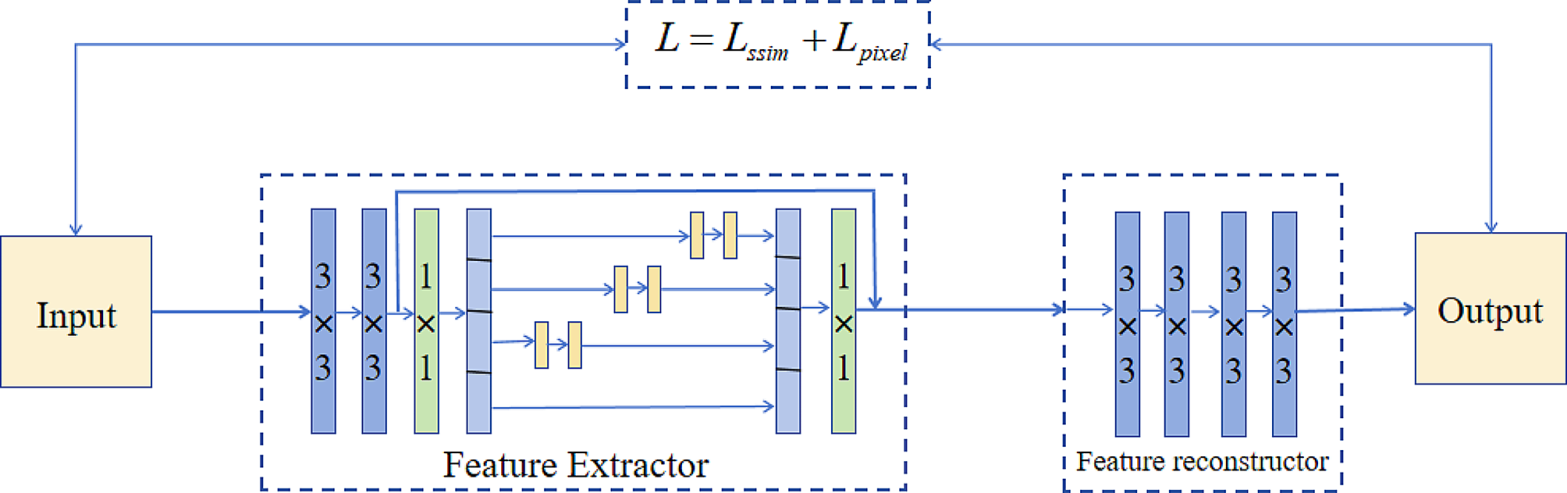
Reconstruction model

During the training process, only the feature extractor and reconstructor are considered, and the fusion layer is not considered. The training model is shown in Fig. 4. When the weight parameters of the training of the feature extractor and reconstructor are fixed, the fusion layer is added to two structures, the multiscale features outputted by the feature extractor are fused, and fusion features are finally inputted to the reconstructor to generate a fusion image. Given that the purpose of training the network is to reconstruct an image, we trained 10,000 CT and MR images and cropped them to 256 × 256 size. In the training parameter setting, learning rate is set at 10^− 4^, and batch size is set at 4. All experiments were conducted on an NVIDIA GeForce RTX 3060 GPU and a 2.10 GHz Intel(R) Core(TM) i7-12700 F CPU, using PyTorch as the compilation environment.

### Fusion result analysis

To validate the effectiveness of the proposed method, we conducted both qualitative and quantitative comparisons with state-of-the-art methods. These methods include DenseFuse, RFN-Nest [36], MSDNet and DFENet [37]. During the evaluation, compared methods should maintain the same resolution as the source images for qualitative and quantitative comparisons.

**Fig. 5:**
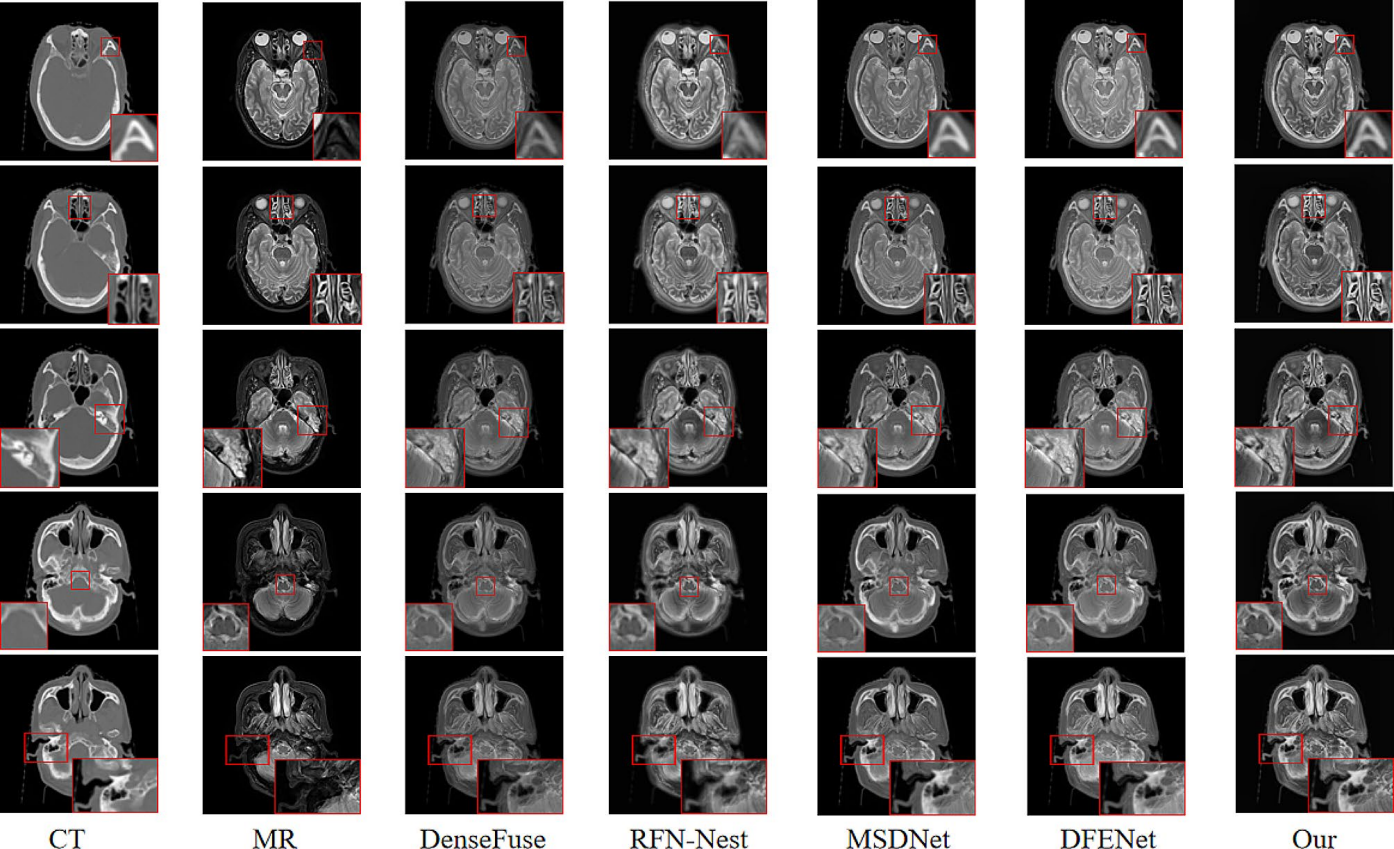
Qualitative comparison: CT and MR images of the first and second columns. The following three columns show the fusion results of the comparison method and the method proposed in this article

1. Qualitative Comparison: A qualitative evaluation was performed by using a patient’s data from the test set. Five pairs of images from different scanning layers of the patient were selected for visual assessment, as shown in Fig. 5. From the images, the proposed method in this paper has two significant advantages over DenseFuse, RFN-Nest, MSDNet and DFENet. First, the fusion results from this paper can preserve the high-contrast characteristics of CT images. This feature is particularly beneficial for diagnosing tumors involving bone invasion because it allows the accurate assessment of tumor boundaries in clinical diagnosis. Fusion results from this paper exhibited clear texture details and structural information with sharp boundaries and minimal information loss.

**Fig. 6:**
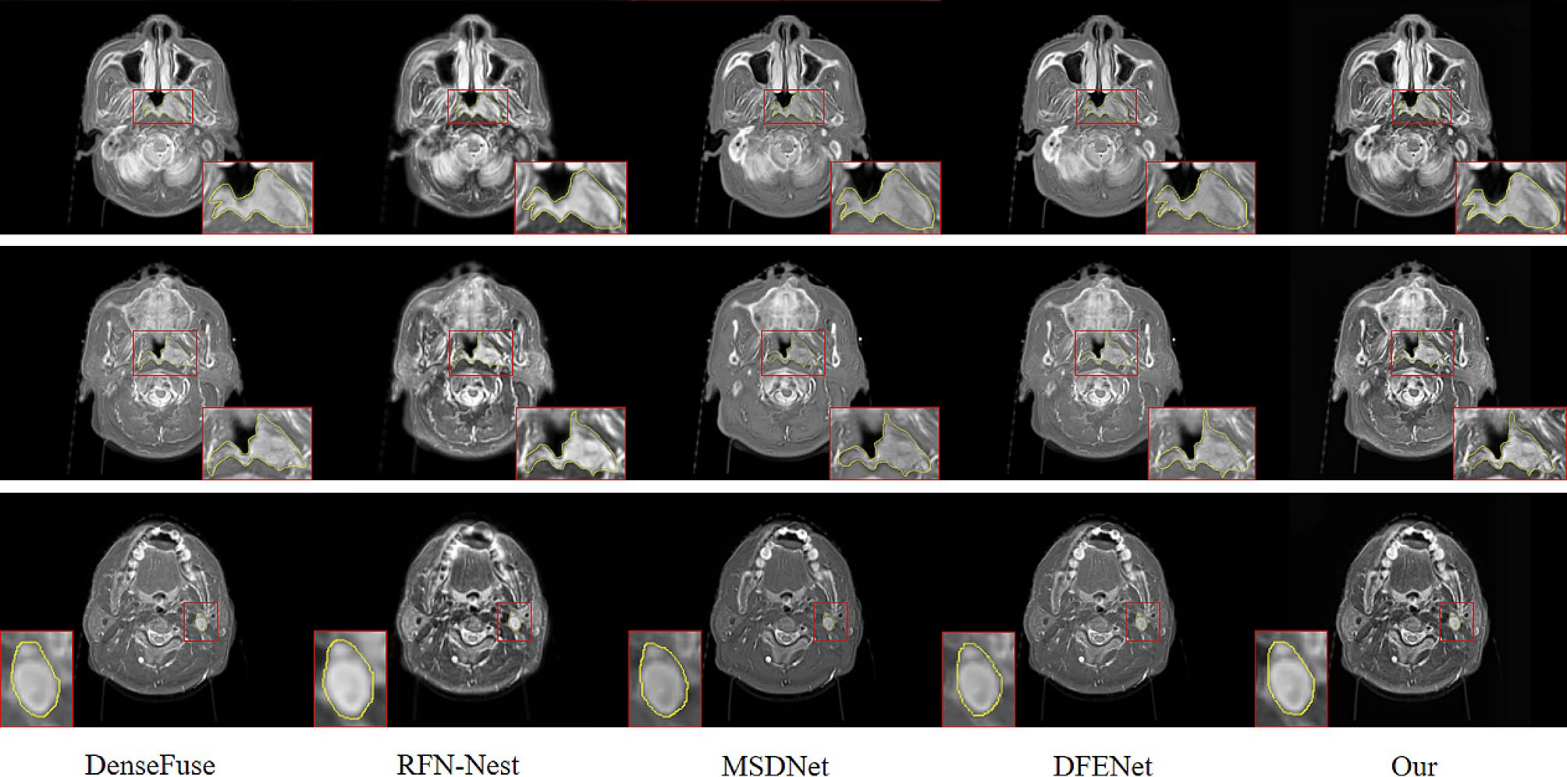
Comparison of target area delineation between tumor and metastatic lymph node lesions

To illustrate that the fusion results from the proposed method aid in tumor delineation by doctors, a senior attending physician with extensive experience conducted a comparison of target delineation on three different scanning layers of a patient. As shown in Fig. 6, the first and second rows depict the delineation of a target area for a patient with nasopharyngeal carcinoma, and the third row represents the boundary delineation of lymph node metastatic lesions in a patients with nasopharyngeal carcinoma. Validated by another senior attending physician, the fusion results from the proposed method can more accurately locate the tumor area boundaries, facilitating precise delineation of the target area.

2) Quantitative comparison: Among the many measurement standards, a single measurement method can only reflect a single feature, and thus we adopted eight indicators to evaluate the fusion image. Methods include average gradient (AG), spatial frequency (SF), entropy (EN), mutual information (MI), peak signal-to-noise ratio, structural similarity index measure (SSIM), visual information fidelity for fusion [38], and quality metric for image fusion [39].

The $AG$ is used to measure the clarity of a fused image. The higher the value of $AG$ is, the higher the image clarity and the better the fusion quality are. Its calculation formula is as follows:

7 $$\eqalign{& AG = {1 \over {(M - 1)(N - 1)}} \cr & \sum\limits_{i = 1}^{M - 1} {\sum\limits_{j = 1}^{N - 1} {\sqrt {{{{{(F(i + 1,j) - F(i,j))}^2} + {{(F(i,j + 1) - F(i,j))}^2}} \over 2}} } } \cr}$$

where $F(i,j)$ represents the pixel values of row $i$ and column $j$ in a fused image, and $M$ and $N$ represent the height and width of an image, respectively.

$SF$ mainly reflects the gray-scale rate of an image. The greater the spatial frequency is, that is, the larger the $SF$ value is, the clearer the image, the clearer the texture and details, and the better the fusion quality are. The calculation formula is as follows:

8 $$SF=\sqrt{{RF}^{2}+{CF}^{2}}$$

$RF$and $CF$ are defined as follows:

9 $$RF=\sqrt{\frac{1}{MN}\sum _{i=1}^{M}\sum _{j=1}^{N}{\left(F\right(i,j)-F(i,j-1\left)\right)}^{2}}$$

10 $$CF=\sqrt{\frac{1}{MN}\sum _{i=1}^{M}\sum _{j=1}^{N}{\left(F\right(i,j)-F(i-1,j\left)\right)}^{2}}$$

where $F(i,j)$ represents the pixel values of row $i$ and column $j$ in a fused image, and $M$ and $N$ represent the height and width of an image, respectively.

$EN$ is mainly a measure of the amount of information contained in a fused image. The amount of information increases with the level of information entropy. Its calculation formula is as follows:

11 $$EN=-{\sum }_{n=0}^{N-1}{p}_{n}{log}_{2}^{{p}_{n}}$$

where $N$ represents the gray level of a fused image and ${p}_{n}$ represents the normalized histogram of the corresponding gray level in the fused image.

$MI$ retains the source image pair information for a fused image. The greater the mutual information is, the more the fused image retains the source image information and the better the fusion quality. The calculation formula is as follows:

12 $$MI=EN\left({\varvec{I}}_{1}\right)+EN\left({\varvec{I}}_{2}\right)-EN({\varvec{I}}_{1},{\varvec{I}}_{2})$$

where $EN(\bullet )$ denotes the information entropy of a computed image, and $EN({\varvec{I}}_{1},{\varvec{I}}_{2})$ denotes the joint information entropy of the image.

$PSNR$ [26] reflects the degree of image distortion by the ratio of the peak power to the noise power of a fusion image. Fusion quality increases with $PSNR$ value, the better the fusion quality. The calculation formula is as follows:

13 $$PSNR=10{lg}^{\frac{{r}^{2}}{MSE}}$$

where $r$ represents the peak value of the fused image and $MSE$ is the mean square error of the difference between a fused image and a source image. $MSE$ is defined as follows:

14 $$MSE(x,y)=\frac{1}{MN}\sum _{i=1}^{M}\sum _{j=1}^{N}{\left(x\right(i,j)-y(i,j\left)\right)}^{2}$$

15 $$MSE=\frac{1}{2}\left(MSE\right({\varvec{I}}_{1},{\varvec{I}}_{f})+MSE({\varvec{I}}_{2},{\varvec{I}}_{f}\left)\right)$$

where ${I}_{1}$and ${I}_{2}$ represents the source image, and ${I}_{f}$ represents the fusion image of ${I}_{1}$ and ${I}_{2}$.

$SSIM$ [26] evaluates the fusion image from three aspects: brightness, contrast, and structure. Structure similarity and fusion quality improves with increasing $SSIM$. The calculation formula is as follows:

16 $$\eqalign{& SSIM(x,y) = \cr & {{\left( {2{\mu _x}{\mu _{y + {c_1}}}} \right)(2{\sigma _{xy}} + {c_2})({\sigma _{xy}} + {c_3})} \over {(\mu _x^2 + \mu _y^2 + {c_1})(\sigma _x^2 + \sigma _y^2 + {c_2})\left( {{\sigma _x}{\sigma _{y + }}{c_3}} \right)}} \cr}$$

17 $$SSIM=\frac{1}{2}\left(SSIM\right({\varvec{I}}_{1},{\varvec{I}}_{f})+SSIM({\varvec{I}}_{2},{\varvec{I}}_{f}\left)\right)$$

where ${\mu }_{x}$ and ${\mu }_{y}$ represent the mean values of $x$ and $y$, respectively, ${\sigma }_{x}$ and ${\sigma }_{y}$ represent the standard deviations of $x$ and $y$, and ${\sigma }_{xy}$ represents the covariance of $x$ and $y$, respectively, ${c}_{1}$, ${c}_{2}$, and ${c}_{3}$ are constants that make the algorithm stable. ${\varvec{I}}_{1}$and ${\varvec{I}}_{2}$ represents the source image, and ${\varvec{I}}_{f}$ represent the fusion image of ${\varvec{I}}_{1}$ and ${\varvec{I}}_{2}$.

$VIFF$ [38] is an index to measure the quality of fused images based on visual fidelity, and fusion ${Q}_{abf}$ [39] is used to measure the performance of significant information of source images in fused images, which can be used in comparing the performance of different image fusion algorithms. The quality of a fused image improves with increasing $VIFF$ and ${Q}_{abf}$.

**Fig. 7:**
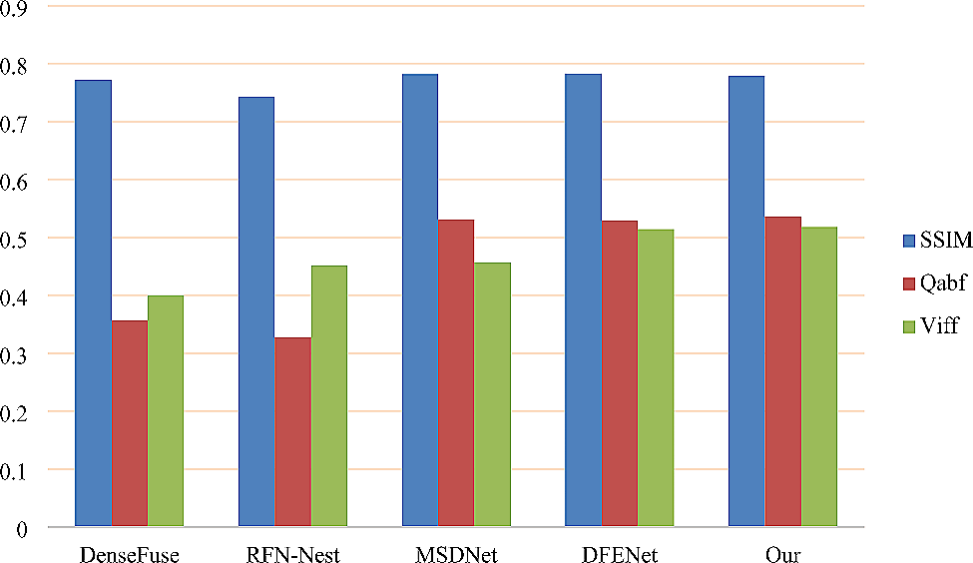
Fusion metric

To further validate the fusion method proposed in this paper, 40 image pairs were selected for quantitative comparison in different scanning layers of patients. $SSIM$, ${Q}_{abf}$, and $VIFF$ all belong to the category of visual perception. The larger the value is, the better the visual effect is compared with the method (Fig. 7). The result of quantitative comparison is the average value of 40 images for each indicator. The specific data are as follows:

**Table 2:** Quantitative results of 40 pairs of MR-CT images

	DenseFuse	RFN-Nest	MSDNet	DFENet	Our	*P* ^a^	*P* ^b^	*P* ^c^	*P* ^d^
AG	3.6152	3.4788	4.5478	4.5792	4.6771	< 0.01	< 0.01	0.01	< 0.01
SF	10.0585	9.5649	13.0434	13.0584	13.2055	< 0.01	< 0.01	0.127	< 0.01
EN	4.3175	4.5693	4.4825	4.4163	4.6039	< 0.01	0.044	< 0.01	< 0.01
MI	1.7306	1.6189	1.8070	1.9137	1.8663	< 0.01	< 0.01	< 0.01	< 0.01
PNSR	19.9831	18.6209	20.3973	19.6427	19.6532	0.048	< 0.01	< 0.01	0.838
SSIM	0.7722	0.7431	0.7815	0.7829	0.7723	0.824	< 0.01	< 0.01	< 0.01
Q_abf_	0.3567	0.3276	0.5311	0.5289	0.5358	< 0.01	< 0.01	< 0.01	0.345
VIFF	0.3999	0.4509	0.4566	0.5141	0.5176	< 0.01	< 0.01	< 0.01	0.394

p^a^, p for DenseFuse versus Our; p^b^, p for RFN-Nest versus Our; p^c^, p for MSDNet versus Our; p^d^, p for DFENet versus Our

Table 2 shows that the proposed method outperforms DenseFuse, RFN-Nest, MSDNet, and DFENet in objective metrics such as AG, SF, EN, while only slightly lagging behind DFENet in the MI metric. However, concerning the SSIM metric, the proposed method slightly trails MSDNet and DFENet. These metrics reflect that the proposed method can preserve gradient information, edge information, and texture details to the maximum extent, reducing spectral distortion and information loss. In terms of visual perceptual evaluation standards, Q_abf_ and VIFF also outperform DenseFuse, RFN-Nest, MSDNet, and DFENet, especially exceeding MSDNet by 0.4% and 6.1% in specific metrics, indicating higher contrast in visual perception categories. Pairwise t-tests were conducted between our method and other methods based on quantitative metrics for a more objective evaluation. From Table 2, it can be inferred that the proposed method exhibits significant differences from the current state-of-the-art methods, with statistical significance.

## Discussion

The imaging signal of CT images for tumors and soft tissues is dark, and distinguishing between lesions and surrounding normal tissues is difficult. MR Images have high resolution and rich soft tissue information, and abundant tumor boundaries and details are greater than those observed in CT images. The fusion of MR and CT images can complement each other’s information, especially for tumors involving bone destruction, such as tumors invading soft tissue and bone at the same time. The observation is more intuitive and accurate, and it helps doctors to quantify, evaluate, and locate pathological tissues clearly [40] and outline the tumor target areas. In a recent study [41], global-local feature extraction strategies and air-frequency fusion strategies are introduced to preserve complete texture details and global contour information. A study [42] proposed a dual-scale zero-learning medical image fusion method based on Res2Net and adaptive guided filtering, utilizing Res2Net to extract deep features. Another study [37] introduced an image fusion method based on a CNN and Transformer, using Res2Net as the backbone framework of the CNN module to enhance local feature extraction. The proposed models utilize Res2Net to capture features at different levels, effectively preserving significant information from source images through multiscale representation. Additionally, a spatial mean attention fusion strategy was designed to adaptively adjust fusion weights for each pixel position, thereby preserving boundary and detail information from source images.

The proposed method was compared qualitatively and quantitatively with the current state-of-the-art methods. Qualitatively, the compared methods exhibited distortions in the fused images, especially evident in DenseFuse and RFN-Nest, where the complete bone information of CT images was not retained, as shown in Fig. 5. While MSDNet and DFENet yielded slightly better results by preserving the soft tissue information of MR images and bone information of CT images, the distinctive features were not prominent upon visual inspection of the fused images. Additionally, it was observed that the fusion results of RFN-Nest were unstable, exhibiting significant artifacts and indistinct features, as highlighted in the fourth column of Fig. 5. Through perceptual comparison, the proposed method was capable of maintaining high contrast in CT images while displaying the soft tissue information of MR images. Compared to DenseFuse, RFN-Nest, MSDNet, and DFENet deep learning methods, the fusion results of our method contained more complete, stable, and prominent feature information. As shown in Fig. 5, the texture details were clearer, and the boundaries were sharper, maximizing the retention of information from the source images and reducing information loss. Furthermore, to demonstrate the superiority of the proposed method, the fusion results of patients at three different scanning layers were delineated. Tumor positions and lymph node metastases of nasopharyngeal carcinoma patients were delineated, as shown in Fig. 6. The first and second rows depict delineations of tumor positions, while the third row illustrates delineations of lymph node metastases. From the delineation results, it can be observed that the delineation results of MSDNet, DFENet, and the proposed method are very close. However, after validation by two senior attending physicians, it was concluded that the delineation results of the proposed method for fused images were the best, enabling more accurate localization of tumor boundaries and facilitating precise delineation of target areas, thus aiding clinicians in providing comprehensive diagnoses.

Quantitatively, to objectively validate the effectiveness of the proposed method, several metrics were selected to evaluate its fused images, as shown in Table 2. From the table, it can be seen that the proposed method performs best in AG, SF, EN, Q_abf_, and VIFF metrics. Compared to the best-performing method, DFENet, the proposed method exhibits improvements of 9.79%, 14.71%, 18.76%, 0.69%, and 0.35%, respectively. This indicates that the proposed method can maximize the retention of gradient information, edge information, and texture details from the source images, thereby reducing spectral loss and information loss. MI and PSNR performance are second best, while SSIM ranks third, indicating that the proposed method retains more structural information from the source images. Furthermore, compared to MSDNet, which also extracts multiscale features, the proposed method outperforms MSDNet in AG, SF, EN, MI, Q_abf_, and VIFF metrics, with only slightly lower scores in PSNR and SSIM. This suggests that the proposed method can extract finer-grained features, making it more advantageous for medical image fusion tasks.

While this study has demonstrated the potential and advantages of medical image fusion, it is important to acknowledge some limitations. For example: (1) The network architecture used in this study is based on fusing single image scan layers rather than addressing the fusion of three-dimensional MR and CT images. This implies that while fusion results for individual scan layers can be obtained, there may be issues of accuracy loss when translating them into overall three-dimensional effects. This limitation could potentially affect the accurate interpretation and diagnosis of medical images. (2) The fusion strategy in this study is specifically designed for the characteristics of intracranial tumor MR and CT images, considering the mean of feature maps. However, this strategy is only applicable to MR and CT images, and may not be sufficiently generalizable to other types of medical images. Therefore, when applied to other diseases or anatomical sites, it may be necessary to redesign or adjust the fusion strategy to accommodate different image features and clinical requirements.

Therefore, in future research, emphasis can be placed on optimizing fusion strategies, including optimizing medical image fusion strategies for different clinical applications. Specific fusion strategies can be designed based on the characteristics of different organs or lesions to improve fusion effectiveness and accuracy in specific target areas. Additionally, a fusion network framework can be designed to fuse original three-dimensional images to further enhance fusion image quality and apply it to clinical practice.

## Conclusion

According to the imaging characteristics of CT and MR images, some fusion methods are ineffective in texture details, boundary contours, and visual quality. An end-to-end MR–CT fusion method based on deep learning is proposed. To retain the significant information of a source image, the feature extractor of the method adopts the Res2Net module to extract multiscale features to ensure the fine granularity of the source image. In addition, the fusion strategy based on spatial mean attention (pixel-level fusion strategy) adopts appropriate fusion weight for each pixel, which can better reflect the effect in details and boundaries. Compared with similar methods, the proposed method achieves the best integration performance in visual subjective evaluation and objective evaluation.

## Data Availability

The datasets used and analysed during the current study are available from the corresponding author on reasonable request.
